# Rush Medical College

**Published:** 1854-03

**Authors:** 


*Rush Medical College.*

The annual session of this Institution for 1853–4, closed on Wednesday the 22nd of February. The term was a very pleasant and successful one. The public commencement was attended by a large audience, and the Valedictory Address, by Professor Blaney, was listened to with great pleasure. The latter will be found in the columns of the present number, and will be read with interest by the Profession. After the public exercises closed, the graduates, the members of the class remaining in the city, and invited guests, repaired to the Sherman House and sat down to an elegant supper, provided for the occasion. After doing justice to the delicacies provided for the physical man, a most animated and pleasant intellectual feast followed. A series of appropriate sentiments and happy responses were kept up until a late hour; thereby adding another to the many proofs that no artificial *stimuli* are necessary to beget or maintain the highest degree of social enjoyment and intellectual repartee. The following are the names of the Graduating Class for the session of 1853–4, with the title of their theses:—

| | |
|---|---|
| J. M. Edwards, | Rubiola. |
| C. W. Davis, | Tires or Milk-Sickness. |
| J. T. Mayfield, | Cholera Morbus. |

| | |
|---|---|
| H. C. Morey, | Effects of the Mind upon the Physical System. |
| W. A. Hillis, | Inflammation. |
| E. Hopkins, | Medical Diploma. |
| R. S. Hallock, | Dysentery. |
| I. N. Davis, | Venesection. |
| J. W. Collver, | Acute Peritonitis. |
| M. W. Robbins, | Inflammation. |
| R. L. Hale, | Phthisis Pulmonalis. |
| T. P. Seller, | Nature and Distribution of the Food of Animals. |
| J. B. Morrison, | Etiology. |
| C. C. Cornett, | Inflammation. |
| S. P. Root, | A Page in Chemistry. |
| David Whitmire, | Mercury. |
| H. W. Man, | Digestion. |
| A. W. King, | Etiology. |
| J. F. Hamilton, | Acute Hepatitis. |
| G. W. Slack, | On Opium. |
| T. D. Fitch, | Hypertrophy. |
| R. M. McArthur, | Origin and Progress of Medical Science |
| J. W. Lynch, | Phthisis Pulmonalis. |
| E. P. Wood, | Fever, its Pathology and Classification. |
| W. Manson, | Miasmatic Fever. |
| W. M. Avery, | Semiology. |
| M. W. Fish, | The Study of Medicine, its Tendency. |
| J. N. B. Elliot, | Pneumonia. |
| Wm. B. Swisher, | Physiology of Generation. |
| Wm. Watson, | Quinia. |
| Reuben Sears, | Scarlatina. |
| S. P. Yeomans, | Contagion. |
| C. D. Watson, | Albumenuria or Bright's Disease. |
| A. Boomer, | Typhoid Fever. |
| J. N. Niglas, | Dissertation on Dropsy. |
| W. Brenton, | Dysentery. |
| H. Fisk, | Scarlatina. |

